# Multilayer network analysis of FMD transmission and containment among beef cattle farms

**DOI:** 10.1038/s41598-022-19981-0

**Published:** 2022-09-20

**Authors:** Chunlin Yi, Qihui Yang, Caterina M. Scoglio

**Affiliations:** Department of Electrical and Computer Engineering, College of Engineering, Kansas State University, Manhattan, KS USA

**Keywords:** Infectious diseases, Infection

## Abstract

As a highly contagious livestock viral disease, foot-and-mouth disease poses a great threat to the beef-cattle industry. Direct animal movement is always considered as a major route for between-farm transmission of FMD virus. Sharing contaminated equipment and vehicles have also attracted increasing interests as an indirect but considerable route for FMD virus transmission. With the rapid development of communication technologies, information-sharing techniques have been used to control epidemics. In this paper, we built farm-level time-series three-layer networks to simulate the between-farm FMD virus transmission in southwest Kansas by cattle movements (direct-contact layer) and truck visits (indirect-contact layer) and evaluate the impact of information-sharing techniques (information-sharing layer) on mitigating the epidemic. Here, the information-sharing network is defined as the structure that enables the quarantine of farms that are connected with infected farms. When a farm is infected, its infection status is shared with the neighboring farms in the information-sharing network, which in turn become quarantined. The results show that truck visits can enlarge the epidemic size and prolong the epidemic duration of the FMD outbreak by cattle movements, and that the information-sharing technique is able to mitigate the epidemic. The mitigation effect of the information-sharing network varies with the information-sharing network topology and different participation levels. In general, an increased participation leads to a decreased epidemic size and an increased quarantine size. We compared the mitigation performance of three different information-sharing networks (random network, contact-based network, and distance-based network) and found the outbreak on the network with contact-based information-sharing layer has the smallest epidemic size under almost any participation level and smallest quarantine size with high participation. Furthermore, we explored the potential economic loss from the infection and the quarantine. By varying the ratio of the average loss of quarantine to the loss of infection, we found high participation results in reduced economic losses under the realistic assumption that culling costs are much greater than quarantine costs.

## Introduction

Foot-and-mouth disease (FMD) is a highly contagious livestock viral disease, which can lead to destructive economic losses^1^. Several major outbreaks have occurred in the UK (2001)^2^, Netherlands (2001)^3^, Japan (2010)^4^, Uganda (2006)^5^ etc. The estimated losses of the FMD outbreak in the UK, 2001 amount to about Pound Sterling 3.1 billion^2^; about 290,000 animals had been culled during the FMD outbreak occurred in Japan, 2010^4^; a study estimated that one FMD outbreak constrained to Kansas US would result in an economic loss varying from US Dollar 43 to 706 million^6^. Therefore, it is of great importance to understand the impacts of different FMD virus transmission routes on the outbreak and design control measures to prevent the spread of the disease in the livestock industries.

Moving infected animals and sharing contaminated equipment are considered two of the most common routes for between-farm infectious disease transmission^7,8^. A number of researches have been conducted to explore the impacts of the different transmission routes on the spread of infectious diseases by building variant between-farm contact networks. Generally, the between-farm transmission routes can be described by two types of contact network—direct contact networks (DCN) which are formed by the movement of livestock between farms, and indirect contact networks (ICN) which are formed by the personnel visits, sharing transport vehicle and tools, etc.^9–12^. Instead of focusing on a single contact network, many studies combined the DCN and ICN into a two-layer network (D&ICN) to analyze the roles each transmission route plays in the disease spreading^8,13–16^. Study^13^ showed that the porcine reproductive and respiratory syndrome virus transmitted by the D&ICN resulted in a larger outbreak compared with the situation when the virus was transmitted by DCN. Sharing haulage vehicles and animal product transport vehicles were found able to increase the contacts between farms by > 50% and accelerate the disease transmission^8^. Study^14^ concluded that the indirect contacts through vehicles and operators were crucial to accurate predictions of the epidemic size. These results all highlighted the impacts of indirect contacts on the disease spread.

Thanks to the rapidly developed communication technology, mitigation strategies based on information sharing^16^ have become increasingly promising. Information sharing was primarily used as a method to improve supply chain resilience to disruptions for livestock production^17,18^. Recently, a number of scholars have used it to control the progressive epidemic^16,19–21^. In study^16^, an agent-based model was developed, where the FMD virus was spread through the D&ICN, the infected farms would inform their partners through the information-sharing network (ISN) and the informed farms would implement preventive measures to suppress the disease spreading. In their study, they assumed that all farms participated in the information-sharing system. However, Farmers’ willingness to participate in the information-sharing system depends on complicated factors such as risk attitudes, privacy, and transparency issues^22,23^. Therefore, efforts are still needed to investigate how participation levels will affect the effectiveness of information-sharing mitigation strategies.

In this paper, we generated a three-layer network including the direct-contact layer (cattle movements), the indirect-contact layer (truck visits), and the information-sharing layer to simulate the FMD virus transmission dynamics among beef-cattle farms in southwest Kansas (SW KS) and the effectiveness of the mitigation strategies based on the information-sharing method. Our goal is to investigate the potential impacts of the virus transmitted by cattle movement (direct contacts) and vehicle visits (indirect contacts) on the FMD outbreaks, and to assess how the information-sharing networks and the fraction of participation farms can affect the epidemic size. To our knowledge, there is no previous study constructing a three-layer network analysis on FMD involving information-sharing techniques. In addition, the influence of different participations in the information sharing are explored unprecedentedly. In this study, hypothetical FMD outbreaks were first simulated on a single-layer DCN (cattle-movement network), a two-layer D&ICN (cattle-movement layer and truck-visit layer), and a three-layer DIC&ISN (cattle-movement layer, truck-visit layer, and information-sharing layer) to assess the effects of each layer on the transmission of FMD virus among beef-cattle farms. We also investigated the impacts of different types of information-sharing layers and participation levels on the epidemic size and quarantine size. Furthermore, a sensitive analysis was conducted to explore the potential economic losses caused by the infection and quarantine to optimize the economy loss and control cost.

## Materials and methods

### Data

We extracted the weekly cattle movement records and the vehicle sharing records between July 1st 2019 and December 31st 2019 from the database^24^. The database provides comprehensive cattle trading records between premises and premises information in SW KS. Cattle trading records includes the identifiers for departure premises and destination premises, truck identifiers for every movement, headcount of cattle carried by truck, and transport date. The premises information includes the premises type (ranches, stockers, and feedlots) and the capacity which is the maximum number of cattle one can hold. In this study, 301 premises in different production type are involved, among which there are 18 ranches, 50 stockers, and 233 feedlots.

### Multilayer network construction

**Figure 1:**
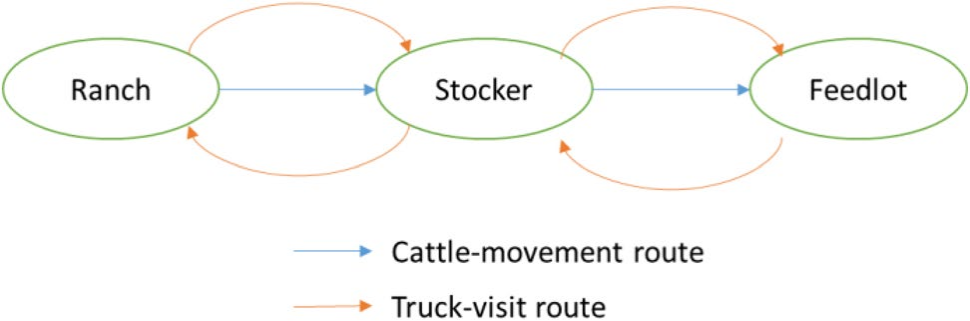
Transmission routes of the FMD virus among different production types of beef-cattle farms.

**Figure 2:**
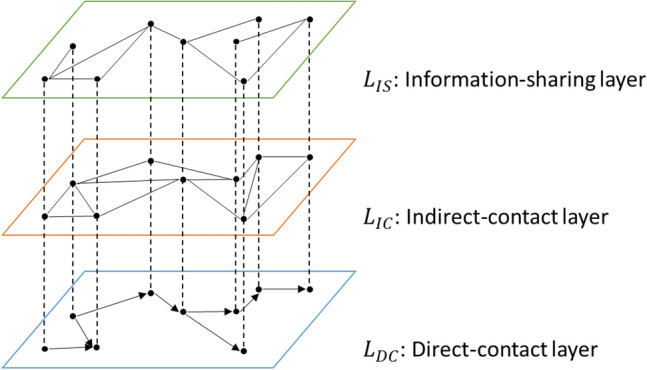
Multilayer network structure of FMD transmission among beef-cattle farms.

To describe the FMD transmission routes by cattle movements and truck visits shown in Fig. 1, we construct a weekly series of weighted two-layer contact network based on the movement data. On top of the contact network, we add an information-sharing layer to simulate the influence of different information-sharing networks and the participation level on suppressing the epidemic. Thus, each network was formed by three independent layers as is shown in Fig. 2. The nodes with the same identifier on each layer represent the same premise; the edges within the layer represents the virus (contact layers) or information (information-sharing layer) transmission routes between premises. Direct-contact layer, indirect-contact layer, and information-sharing layer are presented as ${L}_{DC}=(V, {E}_{DC})$, ${L}_{IC}=(V, {E}_{IC})$, and ${L}_{IS}=(V, {E}_{IS})$, where $V$ represents the set of nodes, and ${E}_{i}$ represents the set of edges within layer $i$. In the direct-contact layer ${L}_{DC}$, every cattle movement is generated as a directed and weighted edge from the departure premise (node) pointing to the destination one, with the weight as the headcount of transported cattle. For the indirect-contact layer ${L}_{IC}$, the premises are connected if they are visited by the same truck in a week. Because the trucks usually return to the departure premises after transporting cattle, the edges within ${L}_{IC}$ are undirected. The weights on ${E}_{IC}$ are the number of visits between premises (“Supplementary information”).

We design three information-sharing layer ${L}_{IS}$ with different network structure but same network density. The first type is an Erdos Renyi random network^25^ with the same network density (0.0068) as the ${L}_{IC}$; the second one is the contact-based network, where the premises share their infection status with their business partners, so ${L}_{IC}^{i-1}$ at week $i-1$ is used as the ${L}_{IS}^{i}$ at week *i*; the third one is based on the geographic distance between premises, where the premises whose distance is shorter than 450 km are connected (with 450 km threshold distance, the distance-based information-sharing network has very similar network density with the other ones). Finally, the infection status of each node on every layer is aggregated to the multilayer network. We construct 26 time-series multilayer networks in total. Each network represents the contact activities in one week, as one week (7 days) is a range of farm-level infection period preceding to detection^8^.

### Network measurements

**Figure 3:**
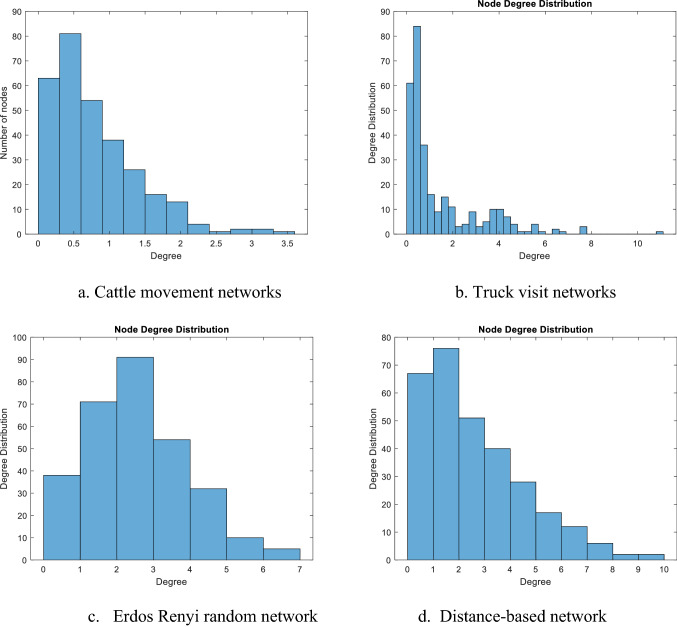
Node degree distributions of different networks.

In order to have an insight into the network topology, we compute some basic measurements which are commonly used to describe the structure of the network. Those measurements include network density which is defined as the ratio of the number of actual edges to the number of potential edges, distribution of node degrees which is defined as the distribution of the number of edges pointing to each node, and the average node degree which is defined as the mean value of the node degrees. Considering that we have 26 homogeneous networks for the cattle movements and the truck visits, we present the mean value of all those measurements over 26 networks. For cattle movement networks, the mean value of the network density is 0.0026, the distribution of the node degrees is presented in Fig. 3a, and the mean value of the average node degree is 0.79. For the truck visits networks, the mean value of the network density is 0.0068, and the distribution of the node degrees is shown in Fig. 3b, and the mean value of the average node degree is 2.04. The network densities and the average node degrees of both types of networks are very small, which mean both cattle-movement networks and the truck-visit networks are sparse. The node degree distributions for cattle-movement networks and the truck-visit networks indicates that there is a small group of nodes with high node degree. For the generated information-sharing networks, the network densities are set close to 0.0068 to keep the sparse characteristics (random network 0.0068 and distance-based network 0.0071); the degree distributions of the Erdos Renyi random network and the distance-based network are shown in Fig. 3c,d.

### Epidemic model

**Figure 4:**
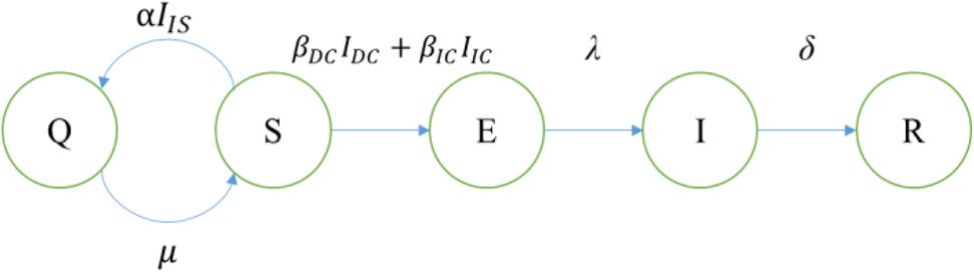
SQEIR model transition process.

To describe the transition process of infection status on each premise, we propose a susceptible-quarantined-exposed-infectious-removed (*SQEIR*) model. In this model, each premise (node) can only be in one of the five states. A susceptible (*S*) node can become exposed (*E*) if it is connected to infectious nodes on ${L}_{DC}$ and ${L}_{IC}$. The transition rate from *S* to *E* is ${\beta }_{DC}{I}_{DC}+{\beta }_{IC}{I}_{IC}$, where ${\beta }_{DC}$ and ${I}_{DC}$ denote the infection rate and the number of contacts to infectious nodes on ${L}_{DC}$, ${\beta }_{IC}$ and ${I}_{IC}$ denote the ones on ${L}_{IC}$. An exposed (*E*) node is not yet infectious, but it will transit to the infectious (*I*) state with rate *λ*. Finally, an infectious node will be removed (*R*) with a removing rate *δ*, which means the cattle in that farm are culled. A susceptible (*S*) node can become quarantined (*Q*) with rate $\mathrm{\alpha }{I}_{IS}$ if it receives notification from infectious neighboring farms on ${L}_{IS}$. The quarantined (*Q*) node can also become susceptible (*S*) at a rate $\mu$. The state transition process is expressed in Eq. (1) and shown in Fig. 4. The parameters used for this model are specified in Table 1; parameters $\mathrm{\alpha }$ and $\mu$ are related to farm owners’ precautionary awareness to FMD. For simplicity, we assume that $\mathrm{\alpha }=1$ and $\mu =0$, which means the informed premises will be quarantined and remain so till the epidemic ends.

1 $$ \begin{aligned}    & S^{\prime}\left( t \right) =  - (\beta _{{DC}} I_{{DC}}  + \beta _{{IC}} I_{{IC}} ) - \alpha I_{{IS}}  + \mu Q \\     & Q^{\prime}\left( t \right) = \alpha I_{{IS}}  - \mu Q \\     & E^{\prime}\left( t \right) = (\beta _{{DC}} I_{{DC}}  + \beta _{{IC}} I_{{IC}} ) - \lambda E \\     & I^{\prime}\left( t \right) = \lambda E - \delta I \\     & R^{\prime}\left( t \right) = \delta I \\  \end{aligned}  $$

**Table 1:** Description and value of the parameters in the model.

Parameter	Description	Value	References
${\beta }_{DC}$	Infection rate of direct contact	0.95	^16^
${\beta }_{IC}$	Infection rate of indirect contact	0.15	^16^
*λ*	Incubation rate	0.83	^16^
$\delta$	Removing rate	0.12	^16^
$\mathrm{\alpha }$	Quarantine rate	1	Assumed
$\mu$	Releasing rate	0	Assumed

The SQEIR model embedded in the multilayer network is simulated in the Generalized Epidemic Modelling Framework (GEMF), which was developed by the Network Science and Engineering group at Kansas State University. GEMF can numerically simulate spreading processes on static multilayer networks^26^, and is available in MATLAB, R, Python, and C programming language. In this work, we further adapt GEMF toolbox in Matlab for simulating disease spreading on time-series multilayer networks.

### Impact of cattle movement, vehicle visits, and information sharing on disease spreading

The hypothetical FMD spreading is simulated on a weekly-series of DCN, D&ICN, and multilayer network with contacts layers and information layer (DIC&ISN). Those different networks are formed by the ${L}_{DC}$, ${L}_{IC}$, and ${L}_{IS}$ we constructed in the previous section. For this simulation, the Erdos Renyi random network is used as the ${L}_{IS}$, and all premises participate in the information sharing system. The FMD virus is seeded in three randomly selected premises in a random week among the first 4 weeks. The simulation is repeated 5000 times. The numbers of the premises in state E, I, and R versus time and the distributions of the final epidemic size (the fraction of the removed premises at the end of the epidemic) of the spread FMD (at least one farm is infected by the seeded premises) are presented and evaluated.

### Impact of different information-sharing layers and premises participation on disease spreading

The idea behind the information sharing network is the following. Suppose that one farm discovers to have cattle infected with FMD. This farm will immediately go to isolation, following the state of Kansas procedure. At this point, this farm will inform its neighboring farms (the farms connected in the network are defined as neighboring farms) of its infection status. The neighboring farms will, in turn, go into a quarantine state, halting all movements of animals and trucks. Cattle in infected farms will be culled, while cattle in farms protected by quarantine, will return to normal conditions once the epidemic is contained. Three different types of the information-sharing layer are added individually to the contact networks. The hypothetical FMD spreading is simulated on the weekly-series of DIC&ISNs with different ${L}_{IS}$—the Erdos Renyi random information-sharing layer, the contact-based information-sharing layer, and the distance-based information-sharing layer. Different premises participations are also considered. We randomly activate 10–100% (with interval 10%) participation nodes and deactivate the rest in each ${L}_{IS}$, and simulate the hypothetical FMD spreading. Considering there are hubs (nodes with higher degree) in both contact networks, we rank the nodes by their node degrees and activate 10–100% (with interval 10%) participation of nodes with the highest degree, and simulate the FMD spread again. All the simulations are repeated 5000 times. The distributions of the final epidemic size and the quarantine size of the different participations are presented and evaluated.

### Sensitivity analysis of the potential economic loss from the epidemic and quarantine

According to study^1^ the FMD infection can cause losses by reducing milk production, suppressing growth rate of livestock, and culling the infected cattle. Considering that quarantined farms stop contact with other farms, which leads to economic losses as well, we proposed a loss function *l* = *xR* + *yQ* (*x* > *y*)*,* where *x* is the average loss for one removed farm, *y* is the average loss for one quarantined farm, *R* is the number of the removed farms, and *Q* is the number of the quarantined farms. To qualitatively analyze the potential loss from the epidemic and the quarantine, we rewrite the loss function as $\overline{l }=R+\overline{y }Q$, where the relative loss per quarantined farm $\overline{y }=y/x$ , and the relative total loss $\overline{l }=l/x$. We vary the value of $\overline{y }$ from 0 to 0.5 with step increment 0.05, and calculate $\overline{l }$ of each ${L}_{IS}$ with different participation levels.

## Results and discussion

### Impact of cattle movement, vehicle visits, and information sharing on disease spreading

**Figure 5:**
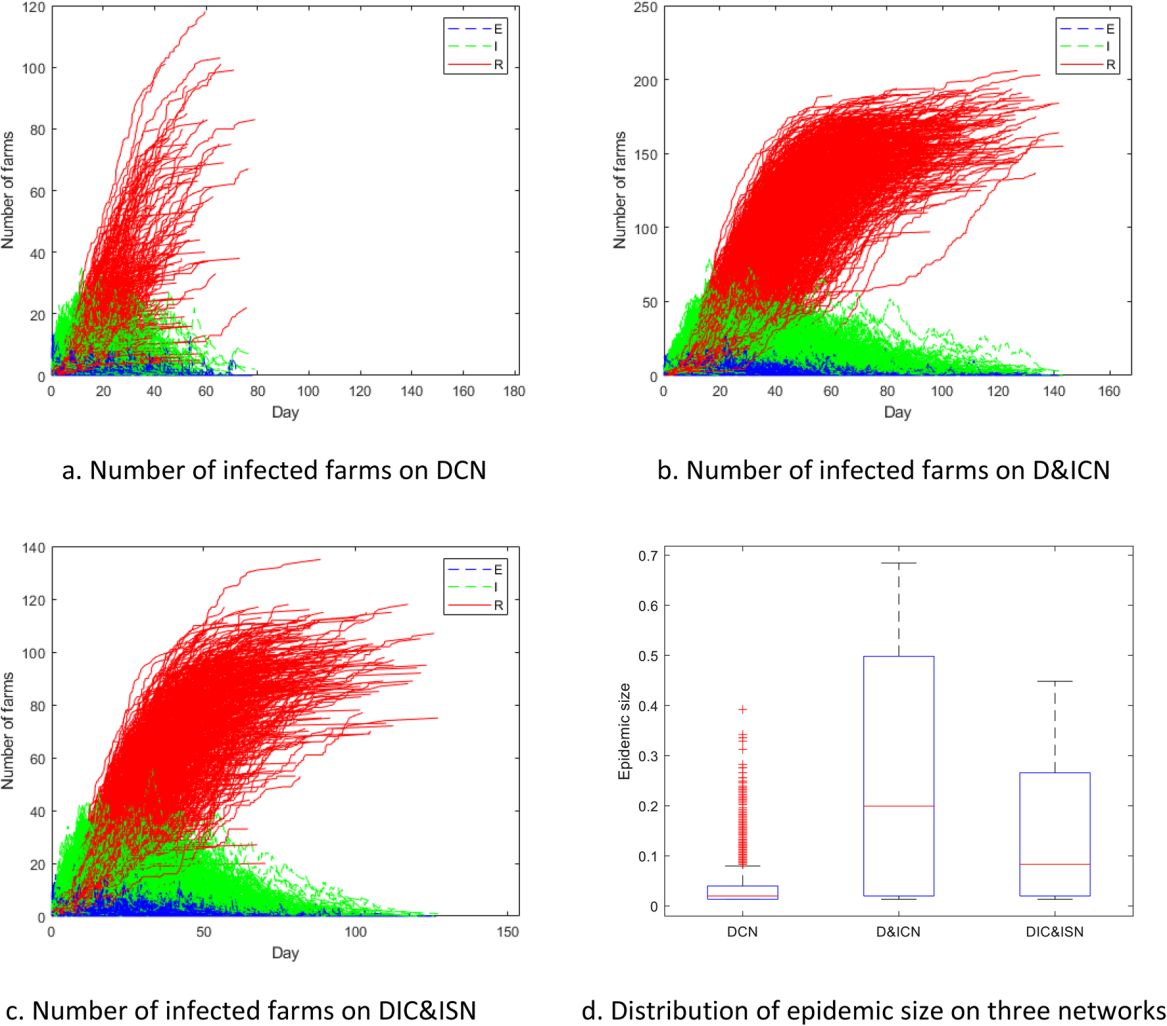
Number of farms in infected compartments and distribution of epidemic size.

The numbers of premises in different infection compartments and the distribution of the epidemic size based on three networks with different layers are shown in Fig. 5. The blue dash line, green dash line, and red solid line represent the number of premises in state E, I, and R versus time separately; the boxplots shows the distributions of the final epidemic size, where the red line in the box represents the median; the upper and lower edges of the box represent the 75% and 25% quartiles; the short lines outside the box are the adjacent (maximum and minimum without outliers); red plus signs are the outliers.

For the hypothetical FMD spreading simulations, the number of simulations producing additional infections on different networks are 1628 (32.56%) on DCN, 1799 (35.98%) on D&ICN, and 1770 (35.40%) on DIC&ISN. The reason for this low outbreak probability is the sparsity of the contact layers, in which there are often no links to or from the initial infected farms during simulation. Surprisingly, the increment of the outbreak probability (the fraction of simulations producing additional infections) on D&ICN from DCN is only 171 (3.42%) even if the average network density of truck-visit network is more than twice of the cattle-movement network. Two facts should be considered here. On the one hand, the indirect infection rate of the truck visit layer is much smaller than the direct infection rate. On the other hand, the number of truck visits is smaller than the number of cattle transported (normally several cattle transported by one truck), which means the weight of the truck visit layer is smaller than that of the cattle movement layer. Therefore, the pathogens are more likely to be transmitted by the cattle movements when the number of infections is small.

For those spread FMD, the average epidemic duration is 4.44 weeks on DCN (minimum 2 weeks and maximum 12 weeks), 8.29 weeks on D&ICN (minimum 2 weeks and maximum 22 weeks), and 6.89 weeks on DIC&ISN (minimum 2 weeks and maximum 18 weeks). As shown in Fig. 4d, the maximum and average epidemic size of spread FMD are 0.392 (118/301) and 0.040 (12/301) on DCN, 0.684 (206/301) and 0.261 (78.5/301) on D&ICN, and 0.449 (135/301) and 0.138 (41.6/301) on DIC&ISN. Our results show that the truck-visit layer not only prolong the average epidemic duration by 86%, but also enlarge the overall epidemic size by 554% on average and 74% on the maximum. Apparently, the truck visits greatly boost the disease transmission. Truck visits become increasingly effective for FMD transmission as the number of infected farms is increasing, although the infection rate and the weight for it are relatively small. The other important characteristics of the truck visit network like higher density and undirected links also contribute to spreading the disease to more farms. The random information-sharing layer has apparent impact on mitigating the epidemics of D&ICN with 17% shorter average duration and 47% smaller average epidemic size.

### Impact of different information-sharing layers and premises participation on disease spreading

**Figure 6:**
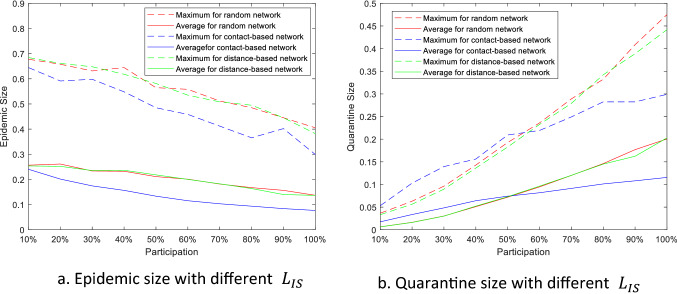
The epidemic size and quarantine size versus random participation.

Figure 6 shows the maximums and average of the epidemic size and quarantine size versus participations when different ${L}_{IS}$ are applied. In this scenario, the participated farms are randomly selected. The solid lines in the figure represent the average while the dashed lines are the maximums; the red, blue, and green lines present the data from random ${L}_{IS}$, contact-based ${L}_{IS}$, and distance-based ${L}_{IS}$ separately. In general, the epidemic size decreases and the quarantine size increases when more farms participated in the information-sharing networks. The maximum and average epidemic size of the contact-based ${L}_{IS}$ is the smallest almost under any participation level, which means the contact-based ${L}_{IS}$ has the best performance on containing the disease spread. The quarantine size of the contact-based ${L}_{IS}$ is the largest when the participation is less than 50%, and the smallest when participation is greater than 60%. Based on the Eq. (1), infected farms is promoting the growth of quarantine while the quarantined farms is prohibiting the growth of infection in every three-layer networks. From the results, we can deduct that the contact-based ${L}_{IS}$ is able to make more farms quarantined under lower participation level to prohibit the infection more effectively than other two ${L}_{IS}$, and that the smaller epidemic size of contact-based ${L}_{IS}$ under higher participation level makes the quarantine size small.

**Figure 7:**
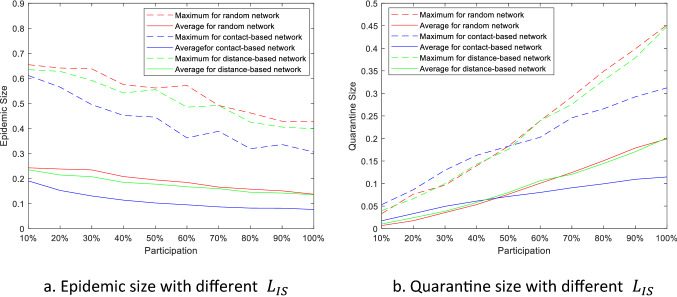
The epidemic size and quarantine size versus highest-degree participation.

In order to more effectively prohibit the disease spreading on the contact networks, we primarily include the farms with the highest node degrees into the information-sharing networks. The epidemic size and the quarantine size are shown in Fig. 7. Compare with the random participation, the performance of contact-based ${L}_{IS}$ on containing epidemic size has an obvious improvement. Under 10% participation, the average epidemic size is 0.19, which is 20.8% decrease from 0.24 with random participation. The average epidemic size decrease to less than 0.1 when participation level is higher than 50%. However, different types of participations have little effect on epidemic size of the other tow ${L}_{IS}$ and the quarantine size of all three ${L}_{IS}$.

### Sensitivity analysis of the potential economic loss from the epidemic and quarantine

**Figure 8:**
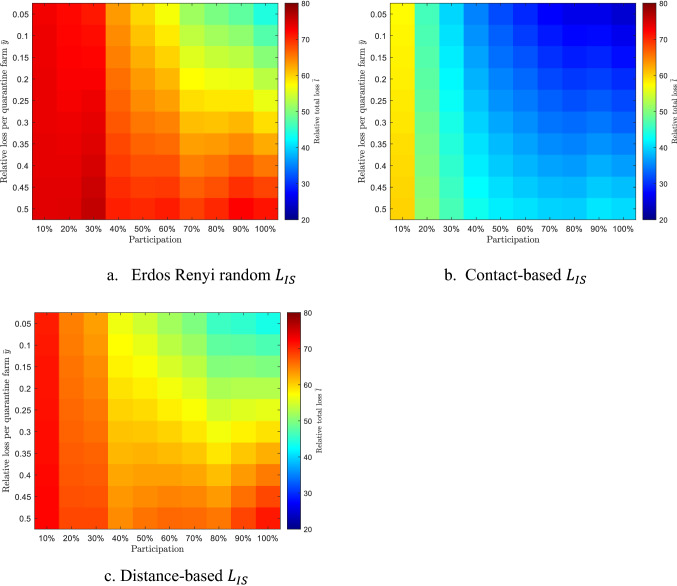
Relative total economic losses on networks with different ${L}_{IS}$ (highest-degree participations).

The potential losses caused by the infection and the quarantine are displayed in Fig. 8. The x-axis represents the participation level ranging from 10 to 100%; y-axis represents the relative loss per quarantine farm $\overline{y }$; the scaled color in each pixel represents the relative total loss $\overline{l }$. Comparing the loss color map of three networks, the relative total loss $\overline{l }$ with the contact-based ${L}_{IS}$ are mostly the smallest under any participation and quarantine loss $\overline{y }$ pair. The smallest economic loss is always achieved in the upper right corner, which suggests that the more farms participated in the information-sharing network, the lower economic losses can be achieved, especially when the quarantine cost is much lower than infection (when $\overline{y }$ is negligible).

**Figure 9:**
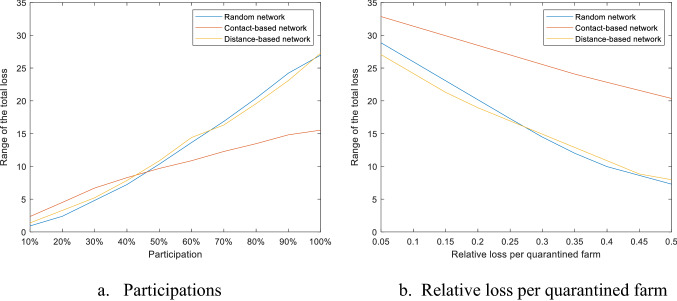
Range of the total loss under different participations and relative quarantine losses.

The results in Fig. 9 show the range of the total loss under different participation or different quarantine loss $\overline{y }$. The range of the total loss is taken as a measurement of the sensitivity between the total loss and the other variables. The ranges of the total loss under 10% participations are less than 3, which means the total loss is not sensitive to the relative quarantine loss $\overline{y }$ under 10% participations. The range increases with the participation level, and decreases with the relative quarantine loss, which means the loss becomes more sensitive to quarantine loss $\overline{y }$ under greater participations, and less sensitive to the participations when the relative quarantine loss $\overline{y }$ grows. From Fig. 7, we know that quarantine size Q is much smaller than epidemic size R when the participation is low, so that R is the dominant term in the loss calculation. The increase rate of the quarantine size is greater than the decrease rate of the epidemic size with participation level based on Fig. 7. The loss decreased from R can be compensated by the increase in Q especially when the relative quarantine loss $\overline{y }$ is large.

## Conclusions

In this study, we firstly conducted hypothetical FMD spreads on a single-layer DCN, a two-layer D&ICN, and a three-layer DIC&ISN, where the results show that the FMD has larger epidemic size and long duration on the two-layer D&ICN than the single-layer DCN, and that the information-sharing layer is able to mitigate the spread of the disease. These results re-addressed the significant influence of the truck visits on the between-farm transmission of FMD virus, and revealed that truck-visit routes can greatly enlarge the outbreak epidemic size, but have limited influence on increasing the outbreak probability. Therefore, the regional animal disease control agencies should take actions to reduce direct-contact rate and control the cattle movements in the early stage of the epidemic to prevent the outbreak. For existing outbreaks, increasing truck sterilization frequency and limiting truck visits are useful policies to mitigate the epidemic size.

The information-sharing network is tested effective for epidemic mitigation. We compared the mitigating performance of three information-sharing networks with different topologies and participation levels. The contact-based network has the best performance on suppressing the epidemic size while maintaining low quarantine size especially when the farms with the highest degrees are participated. For all three information-sharing networks, increased participations result in decreased epidemic sizes but increased quarantine sizes. For contact-based ISN, the epidemic size decreases fastest, while the quarantine size increases slowest. A sensitive analysis is conducted to explore the potential economic losses caused by the infection and quarantine. We found that the losses with contact-based ISN is the smallest under any participation and relative quarantine loss pair, and that an increased participation level generally leads to a deceased economic loss when the quarantine costs are negligible ($\overline{y }<0.3$). The regional agencies could work on calling more farms to participate in the contact-based information-sharing network to ensure that the infection status of the farms is accessible to their business partners. In our results, an 80% participation level is able to display fairly good performance on reducing the economic losses. We focused on the beef-cattle industry in SW KS, but the implement of information-sharing network can be used by the whole livestock industry.

In this study, there are several limitations. First, the other indirect contacts, like personnel visits and air-borne are not considered. Another limit is we did not consider the auction market. Removing these limitations can be the subject of the future work, together with the determination of the optimal participation level.

## Supplementary Information

Supplementary Information.

## Data Availability

The datasets used and analyzed during the current study available from the corresponding author on reasonable request.
