# Author Correction: Multilayer network analysis of FMD transmission and containment among beef cattle farms

**DOI:** 10.1038/s41598-022-25505-7

**Published:** 2022-12-05

**Authors:** Chunlin Yi, Qihui Yang, Caterina M. Scoglio

**Affiliations:** Department of Electrical and Computer Engineering, College of Engineering, Kansas State University, Manhattan, KS USA

Correction to: *Scientific Reports* 10.1038/s41598-022-19981-0, published online 20 September 2022

The Author Contributions section in the original version of this Article was incomplete.

“C.Y. programmed the simulations, prepared all figures, and wrote the main manuscript text, Q.Y. helped with modifying the manuscript text. All authors reviewed the manuscript.”

now reads:

“C.Y., Q.Y., and C.S. formulated the model; C.Y., Q.Y., and C.S. analyzed the data; C.Y., Q.Y., and C.S. developed the methodology; C.Y. developed the simulation tool; C.Y., Q.Y., and C.S. analyzed the results and contributed to the discussions; C.Y., Q.Y., and C.S. contributed to the writing. All authors reviewed the manuscript. ”

The original Article has been corrected.

